# Population snapshot of the extended-spectrum β-lactamase-producing *Escherichia coli* invasive strains isolated from a Hungarian hospital

**DOI:** 10.1186/s12941-022-00493-8

**Published:** 2022-02-10

**Authors:** Kinga Tóth, Ákos Tóth, Katalin Kamotsay, Viktória Németh, Dóra Szabó

**Affiliations:** 1 Institute of Medical Microbiology, Semmelweis University, Budapest, Hungary; 2 Department of Bacteriology, Mycology, and Parasitology, National Public Health Center, Budapest, Hungary; 3 Central Microbiology Laboratory, Central Hospital of Southern Pest National Institute of Hematology and Infectious Disease, Budapest, Hungary

**Keywords:** *Escherichia coli*, ST131, C1-M27, Extended-spectrum β-lactamase, *Bla*_CTX-M-27_, *Bla*_CTX-M-15_

## Abstract

**Background:**

This study was carried out to determine the prevalence and the genetic background of extended-spectrum β-lactamase-producing *Escherichia coli* invasive isolates obtained from a tertiary-care hospital in Budapest, Hungary.

**Methods:**

Between October–November 2018, all invasive ESBL-producing *E. coli* isolates were collected from Central Hospital of Southern Pest. The antimicrobial susceptibility testing was performed according to the EUCAST guidelines. The possible clonal relationships were investigated by core genome (cg)MLST (SeqSphere +) using whole-genome sequencing (WGS) data of isolates obtained from Illumina 251-bp paired-end sequencing. From WGS data acquired antimicrobial resistance genes, virulence genes and replicon types were retrieved using ResFinder3.1, PlasmidFinder2.1, pMLST-2.0, VirulenceFinder2.0 and Virulence Factors Database online tools.

**Results:**

Overall, six *E. coli* isolates proved to be resistant to third-generation cephalosporins and ESBL-producers in the study period. Full genome sequence analysis showed that five *E. coli* isolates belonged to the ST131 clone: two to C1-M27 subclade with *bla*_CTX-M-27_ and three to C2/H30Rx subclade with *bla*_CTX-M-15_. One isolate belonged to ST1193 with *bla*_CTX-M-27_. According to cgMLST, all C2/H30Rx isolates formed a cluster (≤ 6 allele differences), while the *bla*_CTX-M-27_-producing C1-M27 isolates differed at least 35 alleles from each other*.* Both C2/H30Rx and C1-M27 ST131 isolates harbored similar antimicrobial resistance gene sets. However, only C2/H30Rx isolates had the *qnrB* and *aac(3)-*IIa. The isolates carried similar extraintestinal virulence gene set but differed in some genes encoding siderophores, protectins and toxins. Moreover, only one C2/H30Rx isolate carried salmochelin siderophore system and showed virotype B. All isolates showed resistance against ceftriaxone, cefotaxime, and ciprofloxacin, and the C2/H30Rx isolates were also resistant to gentamicin, tobramycin, and ceftazidime.

**Conclusions:**

Out of six ESBL-producing *E. coli*, five belonged to the ST131 clone. This study indicates, that the C2/H30Rx and C1-M27 subclades of the ST131 appear to be the dominant clones collected in a Hungarian hospital.

## Background

Recently, among the most worrying multidrug resistant bacteria the burden of disease caused by third-generation cephalosporin-resistant *Escherichia coli* increased the most, in terms of the number of infections and the number of deaths in Europe [1]. Its global spread is associated with the sequence type 131 (ST131) high-risk clonal group. Members of ST131 are primarily extraintestinal pathogenic *E. coli* (ExPEC) harbor various virulence genes that allow them to cause severe extraintestinal infections, including bloodstream infections, urinary tract infections, pneumonia, and neonatal meningitis [2].

*E. coli* ST131 high-risk clones connected with extended-spectrum β-lactamase (ESBL) production and fluoroquinolone resistance causing multidrug-resistant infections belong to the most prevalent clade C associated with *fim*H30. Within C/H30, two subclades were emerging in the 2000s: C1/H30R subclade with fluoroquinolone resistance (FQ-R) and C2/H30Rx subclade with *bla*_CTX-M-15_, and FQ-R [3, 4]. In the late 2000s, C1-M27 clade with *bla*_CTX-M-27_ of C1/H30R became dominant in Asia and showed global distribution like the C2/H30Rx did before [4]. The expansion of the C1 and C2 subclades was driven by the acquisition of CTX-M genes containing IncF plasmids which evolve with the clone. The F2:A1:B- plasmid carrying the *bla*_CTX-M-15_ gene is associated with the C2/H30Rx subclade and the *bla*_CTX-M-27_ encoding F1:A2:B20 plasmid is associated with the C1-M27 subclade [5, 6]. The other recently emerging high-risk clones among ESBL-producing *E. coli* are ST10, ST38, ST69, ST155, ST315, ST405, ST410, ST648, and ST1193 [7–10].

Previous studies have shown that the proportion of third-generation cephalosporin-resistance among invasive *E. coli* has risen in Hungary from 5.1% in 2006 to 22.6% in 2018 [11, 12]. However, there are few data available on the incidence of third-generation cephalosporin-resistant invasive *E. coli* from Hungary, and its genomic epidemiology and no prospective study was made before.

This study was designed to determine the incidence of third-generation cephalosporin-resistant invasive *E. coli* collected from a Hungarian hospital; and to perform genomic typing of these isolates in order to characterize their genetic background.

## Methods

### Study design

Central Hospital of Southern Pest National Institute of Hematology and Infectious Diseases is a tertiary-care hospital in Budapest, Hungary, with a capacity of approximately 1400 beds. Between October–November 2018, all invasive consecutive non-duplicate clinical isolates of ESBL-producing *E. coli* were collected in the Central Hospital of Southern Pest. An isolate was considered invasive if it has been isolated from blood or cerebrospinal fluid [1].

### Bacterial isolates and antimicrobial susceptibility testing

The culturing from clinical samples, the preliminary antibiotic susceptibility tests and the identification of isolates were performed in the clinical microbiological laboratory of the hospital. All isolates were identified using matrix-assisted laser desorption ionization time-of-flight mass spectrometry (MALDI Biotyper, Bruker, Bremen, Germany).

The ability of ESBL-production were determined by Double Disc Synergy Test (DDST) for all isolates. The DDST confirmation test was performed by the “ESβL Detection Disc Set” (MAST Diagnostica, Reinfeld, Germany) according to the manufacturer’s instruction.

The antibiotic susceptibility test of putative ESBL-producing isolates was performed to ceftriaxone, ceftazidime, cefotaxime, gentamicin, amikacin, tobramycin, fosfomycin, ceftazidime/avibactam, tigecycline, ertapenem by MIC Test Strips (Liofilchem, Roseto degli Abruzzi, Italy); and to imipenem, meropenem, ciprofloxacin, colistin by broth microdilution and interpreted using EUCAST guidelines (EUCAST v_8.0; [13], EUCAST v_11.0 [14]). Changes have been made between the clinical breakpoints of EUCAST v_8.0 (valid in 2018) and the current EUCAST guideline v_11.0. The results were interpreted according to both versions. The clinical breakpoints for interpretation are shown in the Table 1.

**Table 1:** Antimicrobial susceptibility of the ESBL-producing *E. coli* isolates (MIC (mg/L)

Antimicrobial agent		Isolate						MIC breakpoint (mg/L), 2018		MIC breakpoint (mg/L), 2021	
		Ec1	Ec2	Ec3	Ec4	Ec5	Ec6	S ≤	R >	S ≤	R >
Cephalosporins	CRO	256	256	256	256	256	256	1	2	1	2
	CAZ	32	32	32	4	4	4	1	4	1	4
	CTX	≥ 256	≥ 256	≥ 256	64	64	≥ 256	1	2	1	2
	CZA	1	0,5	1	0.5	0.25	0.125	8	8	8	8
Carbapenems	ETP	0.125	0.125	0.125	0.008	0.008	0.016	0.5	1	0.5	0.5
	MEM	0.064	0.125	0.064	0.032	0.032	0.032	2	8	2	8
	IMI	0.125	0.125	0.125	0.125	0.125	0.125	2	8	2	4
Fluoroquinolone	CIP	256	64	64	64	64	16	0.25	0.5	0.25	0.5
Aminoglycosides	GM	16	64	64	2	1	0,5	2	4	2	2
	AK	2	4	2	4	4	16	8	16	8	8
	TM	8	32	8	1	2	1	2	4	2	2
Tetracycline	TGC	0.25	0.5	1	0,25	1	0.125	1	2	0.5	0.5
Miscellaneous agents	COL	0.125	0.25	0.125	0.25	0.25	0.064	2	2	2	2
	FOS	0.25	1	4	2	4	1	32	32	32	32

The used antibiotics: ciprofloxacin (CIP), ceftriaxone (CRO), ceftazidime (CAZ), cefotaxime (CTX), ceftazidime/avibactam (CZA), gentamicin (GM), amikacin (AK), tobramycin (TM), tigecycline (TGC), fosfomycin (FOS), colistin (COL), ertapenem (ETP), meropenem (MEM), imipenem (IMI). The EUCAST MIC clinical breakpoints (EUCAST v_8.0 and EUCAST v_11.0) were given for interpretation. All aminoglycosides MIC breakpoint was added for indication *E. coli* systemic infections. The MIC breakpoint value of ceftriaxone and cefotaxime represent indications other than meningitis

The susceptibility tests were performed using Mueller Hinton Agar (Bio-Rad, Hercules, California, USA) and the cultures were incubated at temperatures of 35–37 °C for 16–20 h.

### Statistical analysis

The incidence rate or incidence density of invasive infection caused by *E. coli* were calculated per 1000 hospital admissions or 1000 patient–days, respectively.

### Molecular characterization

The DNA extraction from the bacterial cultures was performed with the DNeasy UltraClean Microbial Kit (Qiagen, Hilden, Germany) according to the manufacturer's instruction. Genomic DNA analysis of the isolates was performed by whole-genome sequencing (WGS) using Illumina MiSeq 251-bp paired-end sequencing. Raw data were processed by Ridom SeqSphere + and assembly was performed using Velvet 1.1.04 [15]. The quality of the sequencing of a given sample was accepted if the genome quality indicators of the assembly were: N50 > 100 kb, approximated assembled genome size: 5 ± 0.3 Mb and average depth of sequencing coverage at least 50-fold. The possible clonal relationships were investigated by core genome (cg)MLST (SeqSphere +). Cluster type (CT) for *E. coli* was defined as isolates attributed to the same clone with ≤ 10 differing alleles (CT threshold) within a group. The ST131 isolates were compared to three international ST131 *E. coli* genomes (C1-M27: EC81009 [16] and H105 [17], C2/H30Rx: JJ2434 [18]) obtained from GenBank, to reveal the relatedness to the subclades of the ST131 clone. The replicon types were retrieved using PlasmidFinder2.1 and pMLST-2.0 [19] online tools at the Center for Genomic Epidemiology (CGE, http://genomicepidemiology.org/). In order to determine sequence similarity (coverage and identity), the isolates were aligned to the pEC-81009 (125.7 Kb, Group 1 replicon: IncFII_1, IncFIA_2, IncFIB_20), to the uk_P46212 plasmid (143.7 Kb, Group 2 of replicon: IncFII_2, IncFIA_1) and pU1 (F1:A1:B16) reference plasmid sequences [6]. The reads were trimmed (the quality threshold of 30 reads was filtered for a window of 20 bases based on filtering) and mapped to the reference plasmid sequences by BWA (SeqSphere +) and the mapped sequences were analyzed by BLASTn. The pEC-81009 (C1 clades reference plasmid, Group 1 of replicon: IncF[F1:A2:B20]; CP021180), the uk_P46212 plasmid (C2 clades reference plasmid, Group 2 of replicon: IncF[F2:A1:B-]; CP013657), and the pU1 (MK295825) reference plasmid sequences, and their terminologies and definitions were obtained from a meta-analysis of Kondratyeva et al. [6], and the GenBank. The presence of the M27PP1 prophage-like region characteristic was investigated by comparing it with the M27PP1 prophage-like region sequences of KUN5781 [20]. From WGS data the acquired antimicrobial resistance genes were identified using ResFinder3.1 [21] CGE online tool. The online analysis was performed with the default settings of 30% of the identity threshold and 20% of minimum length overlap. The presence of gene was accepted when the identity and coverage were > 90% identified.

Virulence factor genes were retrieved using VirulenceFinder2.0 [22] and Virulence Factors Database (VFDB [23]) online tools. The virotypes A to D of the ST131 isolates were assigned according to the scheme developed according to Blanco et al. [24]: virotype A *(afa* positive, *iroN* negative, *ibeA* negative, *sat* positive or negative), virotype B (*afa* negative, *iroN* positive, *ibeA* negative, *sat* positive or negative), virotype C (*afa* negative*, iroN* negative, *ibeA* negative, *sat* positive), and virotype D (*afa* negative, *iroN* positive or negative, *ibeA* positive, *sat* positive or negative).

The raw reads are available on the Sequence Read Archive (SRA) database under the BioProject number PRJNA683640.

## Results

### Clinical epidemiology

Twenty-five *E. coli* were isolated from haemoculture in the study period. Six from 25 isolates (24%) proved to be resistant to third-generation cephalosporins and were obtained from patients with bloodstream infection. The incidence density and the incidence rate of third-generation cephalosporin-resistant *E. coli* were 0.11 per 1,000 patient-days and 0.79 per 1,000 hospital admission, respectively. The phenotypic detection tests showed that all of them were ESBL-producer. Out of the six isolates collected from the Central Hospital of Southern Pest, two isolates originated from the Haematology and Stem Cell Transplantation ward, two from the Infectious Diseases ward, one from the Intensive Care Unit and the last one from the 1st Internal Medicine ward. The median age of the patients was 70 years (range 38–90), and the sex ratio was 1:1.

### Antimicrobial susceptibility

According to the both EUCAST guideline versions the isolates were resistant to ceftriaxone, cefotaxime, and ciprofloxacin but remained susceptible to colistin, fosfomycin, ceftazidime-avibactam, and carbapenems (Table 1). The Ec1, Ec2, and Ec3 showed resistance to ceftazidime, gentamicin, and tobramycin too. Among several revised clinical breakpoints of investigated antibiotics only the change of tigecycline breakpoints has affected the susceptibility interpretations of our isolates.

### Molecular characterization

The whole-genome sequencing and genome assembly results met all quality requirements for all isolates. The assembled draft genome size of the isolates was 5 325 328 bp for Ec1, 5 309 727 bp for Ec2, 5 298 859 bp for Ec3, 5 041 503 bp, for Ec4, 5 070 035 bp for Ec5 and 5 115 835 bp for Ec6.

**Fig. 1:**
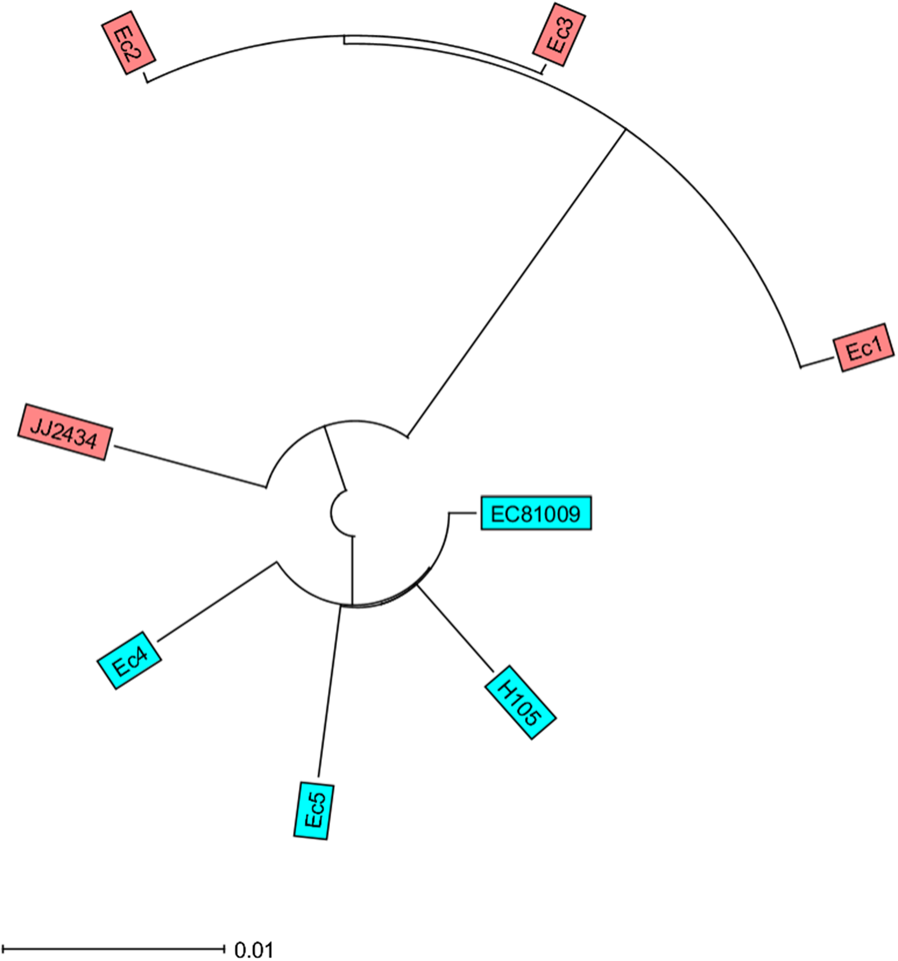
The ST131 subclade-specific phylogenetic relationship of the *Escherichia coli* isolates. The unrooted circular phylogenetic tree of ST131 *Escherichia coli* isolates based on the Ridom™ SeqSphere + core genome multilocus sequencing typing (cgMLST) including 2517 alleles. The blue colour corresponds to Sequence Type 131 C1-M27 subclade, while the red to Sequence Type 131 C2/H30Rx subclade

The six ESBL-producing *E. coli* isolates could be assigned to two different sequence types (ST) by MLST*.* Of the six isolates, five (Ec1-Ec5) belonged to the globally dominant ST131 clone (O25:H4 serotype), which comprised two distinguished groups of strains with different characteristics. The first group contains two isolates with *bla*_CTX-M-15_ and one isolate with *bla*_CTX-M-15_ and *bla*_TEM-1B_ while the two other isolates harbored *bla*_CTX-M-27_ ESBL-genes. The remaining isolate belonged to the ST1193 with *bla*_CTX-M-27._ Analysis of genetic relatedness between the strains was performed by cgMLST. The five ST131 isolates were compared to one C2/H30Rx (JJ2434) and two C1-M27 (H105, EC81009) international ST131 ESBL-producing *E. coli* isolates, to determine the subclade-specific relatedness of the isolates within the ST131 (Fig. 1). The JJ2434 isolate and the three CTX-M-15-producing isolates (named the first group below) belonged ST131 C2/H30Rx subclade. The H105 and EC81009 isolates with the two CTX-M-27-producing isolates from this study belonged to the ST131 C1-M27 subclade. The Ec4 and Ec5 harboured exclusively the C1-M27 clade-specific M27PP1 prophage-like genomic island.

**Fig. 2:**
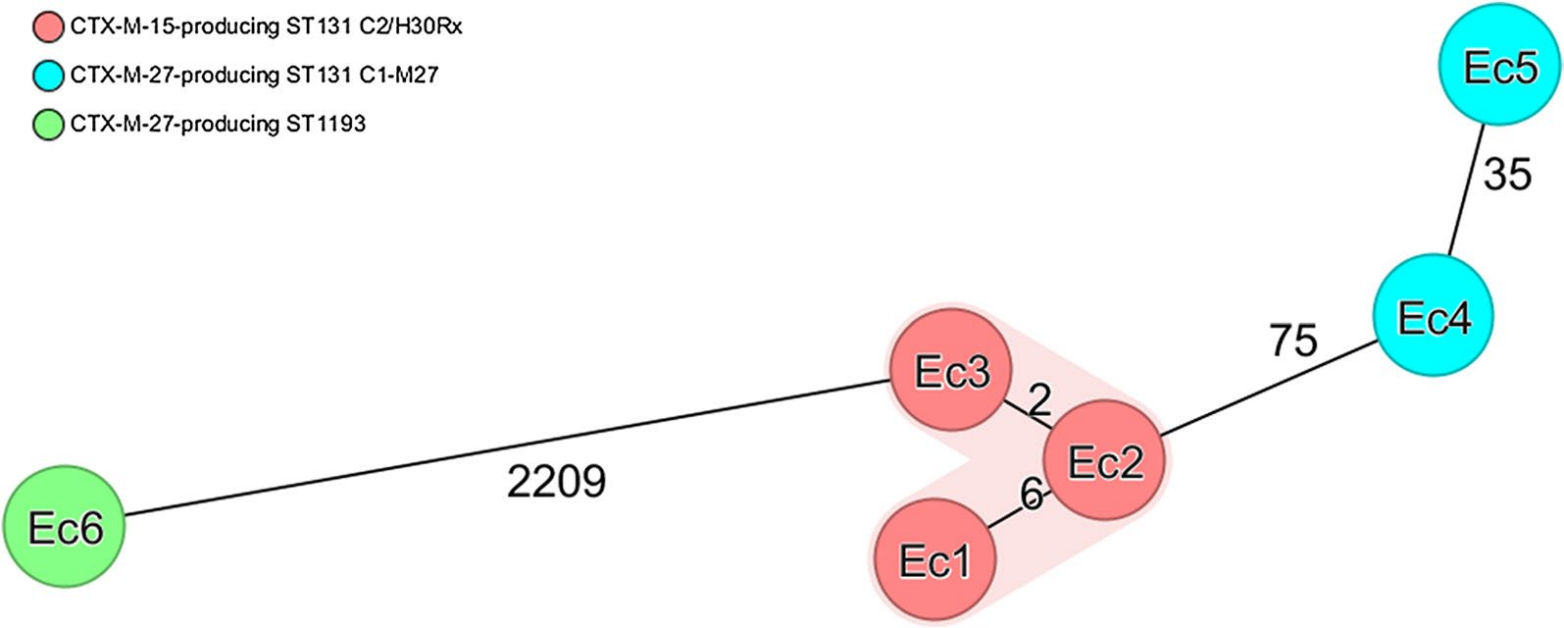
Core genome MLST based Minimum Spanning Tree of the ESBL-producing *Escherichia coli* isolates. Minimum spanning tree of *Escherichia coli* isolates based on the Ridom™ SeqSphere + core genome multilocus sequencing typing (cgMLST) including 2517 alleles with a cluster threshold ≤ 10. Colors correspond with a different genotype characteristic and sequence type of the group of strains. Numbers of allele differences are indicated between the two nodes

According to cgMLST (Fig. 2), all the three Hungarian ST131 *E. coli* isolates producing *bla*_CTX-M-15_ formed a close cluster (≤ 6 allele differences), while the C1-M27 isolates producing *bla*_CTX-M-27_ differed at 35 alleles from each other. The ST1193 isolates showed ≥ 2209 allele differences from any others.

The isolates of each group belonged to ST131 harboured very similar resistance gene sets. The characteristics of resistome and plasmid replicon types of the isolates are shown in Table 2. Although, there are some differences between the two ST131 subclades in terms of resistance genes like the *qnrB* (FQR*)* and *aac(3)-IIa* (aminoglycoside-R) which were present only in C2/H30Rx isolates. The ST1193 isolate harboured L416F mutation in *parE*, and also had no *qnrB* and *aac(3)-IIa* genes. Three plasmid families were detected: IncF, IncQ, and Col-like. The IncF replicons were present in every isolate, but only the C1-M27 ST131 isolates (Ec4, Ec5) carried the Group1 replicons (IncFII_1, IncFIA_2, IncFIB_20). The coverage of the Group1 (F1:A1:B20) reference plasmid by the reads of Ec4 and Ec5 was 96%, and 72%, respectively (Table 3). None of the C2/H30Rx isolates (Ec1-Ec3) co-harboured the two reference replicons of Group2 (IncFII_2, IncFIA_1). The Ec2 did not carry any IncFIA replicon but carried the IncFII_2 replicon. The coverage of the Group2 (F2:A1:B-) reference plasmid by the reads of Ec1, Ec2, and Ec3 was 70%, 47%, and 70%, respectively. The reads of Ec1 and Ec3 covered pU1 plasmid to a higher breadth compared to Group2 reference (≥ 91% vs. 70%). The ST1193 isolates harbored IncFII_4-like, IncF1A_1, IncFIB_10, and the repCol156, and repCol(BS512) replicons. The Col(pHAD28), Col156, and Col8282 replicons were at least present in one of the ST131 isolates.

**Table 2:** Resistome and plasmid replicons of the ESBL-producing ExPEC strains

Isolate		Ec1	Ec2	Ec3	Ec4	Ec5	Ec6
β-lactams	*bla*CTX-M-15	+	+	+			
	*bla*CTX-M-27				+	+	+
	*bla*TEM-1B		+				
Fluoroquinolones	gyrA* (S83L)	+	+	+	+	+	+
	gyrA* (D87N)	+	+	+	+	+	+
	parC* (S80I)	+	+	+	+	+	+
	parC* (E84V)	+	+	+	+	+	
	parE* (I529L)	+	+	+	+	+	
	parE* (L416F)						+
	*qnrB19*^*‡*^	+	+	+			
MLS	*mph(A)*	+		+	+	+	+
	*mdf(A)*	+	+	+	+	+	+
Sulphonamide	*sul1*	+	+	+	+	+	+
	*sul2*	+	+	+	+	+	+
Tetracyclines	*tet(A)*	+	+	+	+	+	+
Trimethoprim	*dfrA17*	+		+	+	+	+
	*dfrA1*		+				
Aminoglycosides	*ant(3'')-Ia*		+	+			
	*aadA1*		+				
	*aadA5*	+		+	+	+	+
	*aph(3'')-Ib*	+	+	+	+	+	+
	*aph(6)-Id*	+	+	+	+	+	+
	*aac(3)-IIa*	+	+	+			
Plasmid replicon types		IncFIB IncFIA IncFII Col156 Col(pHAD28)	IncFIB IncFII IncQ1 Col8282 Col(pHAD28)	IncFIB IncFIA IncFII Col156 Col(pHAD28)	IncFIB IncFIA IncFII Col156 Col8282 Col(pHAD28	IncFIB IncFIA IncFII	IncFIB IncFIA IncFII Col156 Col(BS512)

Acquired antibiotic resistance genes, resistance related point mutations of the isolates. The Ec1, Ec2, Ec3 correspond to ST131 C2/H30Rx isolates, Ec4, Ec5 correspond to ST131 C1-M27 isolates and Ec6 corresponds to ST1193. The + indicates the presence of a gene, *the mutations in quinolone resistance determining region, and ^‡^the plasmid-mediated quinolone resistance

**Table 3:** The coverage and identity of the ST131 isolates and the reference plasmids

Isolates	ST131 subclade	Reference plasmid sequence	Coverage (%)	Identity (%)
Ec1	C2/H30Rx	Group2	70	97.89
		pU1	91	99.29
Ec2	C2/H30Rx	Group2	47	99.20
		pU1	52	96.67
Ec3	C2/H30Rx	Group2	70	97.21
		pU1	92	99.87
Ec4	C1-M27	Group1	96	99.93
Ec5	C1-M27	Group1	72	99.83

The Group1 plasmid (135.7 Kb) refers as CTX-M-27-encoding IncF[F1:A2:B20] plasmids, Group2 (143.7 Kb) plasmid as CTX-M-15-encoding IncF[F2:A1:B-] plasmids, and pU1 (144 Kb) as IncF[F1:A1:B16] plasmid. The Ec1, Ec3, Ec3 correspond to ST131 C2/H30Rx isolates, Ec4, Ec5 correspond to ST131 C1-M27 isolates and Ec6 corresponds to ST1193

Table 4 shows the virulence gene sets and corresponding virotype. The virulome of the isolates belonged to ST131 showed high similarities. These isolates belonged to two virotypes: B and C. Four ST131 isolates (Ec1, Ec3-5) showed virotype C and the Ec2 ST131 isolate showed virotype B. The Ec2 carried 63 virulence genes while the other isolates (including the ST1193 isolate) carried 57 ± 1 virulence genes. Difference was found between the iron uptake genes (Ec2 carried exclusively the *iroB,iroC, iroD, iroE, iroN* genes) and several toxin genes.

**Table 4:** Virulome of the ESBL-producing ExPEC strains

Virulence genes		Isolate					
		Ec1	Ec2	Ec3	Ec4	Ec5	Ec6
Adhesins	*fimA*	+	+	+	+	+	+
	*fimB*	+	+	+	+	+	+
	*fimC*	+	+	+	+	+	+
	*fimD*	+	+	+	+	+	+
	*fimE*	+	+	+	+	+	+
	*fimF*	+	+	+	+	+	+
	*fimG*	+	+	+	+	+	+
	*fimH*	+	+	+	+	+	+
	*fimI*	+	+	+	+	+	+
	*sfaX*	+	+	+	+	+	+
	*mat operon*	+	+	+	+	+	+
	*iha*	+	+	+	+	+	+
	*crl*	+	+	+	+	+	+
	*csg operon*	+	+	+	+	+	+
Invasine	*aslA*	+	+	+	+	+	+
	*kpsD*	+	+	+	+	+	+
	*kpsM*	+	+	+	+	+	+
	*kpsT*						+
	*ibeB*	+	+	+	+	+	+
	*usp*	+	+	+	+	+	+
Iron uptake	*chuA*	+	+	+	+	+	+
	*chuS*	+	+	+	+	+	+
	*chuT*	+	+	+	+	+	+
	*chuU*	+	+	+	+	+	+
	*chuV*	+	+	+	+	+	+
	*chuW*	+	+	+	+	+	+
	*chuX*	+	+	+	+	+	+
	*chuY*	+	+	+	+	+	+
	*sitA*	+	+	+	+	+	+
	*sitB*	+	+	+	+	+	+
	*sitC*	+	+	+	+	+	+
	*sitD*	+	+	+	+	+	+
	*fyuA*	+	+	+	+	+	+
	*iutA*	+	+	+	+	+	+
	*iucA*	+	+	+	+	+	+
	*iucB*	+	+	+	+	+	+
	*iucC*	+	+	+	+	+	+
	*irp2*	+	+	+	+	+	+
	*iroB*		+				
	*iroC*		+				
	*iroD*		+				
	*iroE*		+				
	*iroN*		+				
Protectins/serum resistance	*ompT*	+	+	+	+	+	+
	*iss*	+	+	+	+	+	
	*Bor*	+	+	+	+	+	
	*gad*	+	+	+	+	+	+
	*traT*	+	+	+	+	+	+
	*traD*	+	+	+	+	+	
	*neuA*						+
	*neuC*						+
Toxins	*astA*	+	+	+			
	*sat*	+	+	+	+	+	+
	*vat*						+
	*senB*	+		+	+		+
	*hlyF*		+				
Virotype		C	B	C	C	C	NA

The table includes all virulence genes present in at least one isolate. The + indicates the presence of a gene. NA: not applicable. The Ec1, Ec3, Ec3 correspond to ST131 C2/H30Rx isolates, Ec4, Ec5 correspond to ST131 C1-M27 isolates Ec6 corresponds to ST1193 isolate

## Discussion

In our study, the incidence density of invasive infection caused by third-generation cephalosporin-resistant *E. coli* was 0.11 per 1,000 patient-days and the incidence rate was 0.79 per 1,000 hospital admission. There are no previous data on the incidence of third-generation cephalosporin resistant *E. coli* invasive infections in Hungary.

De Kraker et al. investigated changes in the trends of blood stream infections for major bacterial pathogens by analysing the European Antimicrobial Resistance Surveillance System database between 2002–2008. In 2008 the average incidence density of invasive infections caused by third-generation cephalosporin resistant *E. coli* was 0.03/1,000 patient-days in Europe. There was significant difference in incidence density between northern region and southern region of Europe: 0.002/1,000 patient-days and 0.06/1,000 patient-days, respectively. In the eastern region of Europe (including Hungary) the incidence density was similar to the average (0.02/1,000 patient-days) [25]. These findings supported by investigation of Martelius et al. where the incidence density was 0.008/1,000 patient-days in 17 Finnish acute care hospitals [26] and investigation of De Angelis et al. where the incidence density was 0.1/1,000 patient-days in a teaching hospital in Rome, Italy [27] in similar period. The incidence density found in our study has increased significantly compared to incidence density found in eastern region of Europe in 2008 and became similar to that in the study from Rome.

In our study, six from the 25 *E. coli* isolates (24%) proved to be third-generation cephalosporin-resistant and ESBL-producer. In 2010, Pál T. et al. found 26 ESBL-producers among 117 *E. coli* isolates (22.2%) from bloodstream infections collected in three university hospitals in Hungary between March and November 2010, where nine of them belonged to ST131 clone carrying exclusively *bla*_CTX-M-15_ [14]. The rate of ESBL-producer *E. coli* was similar in our study, however, the rate of ST131 was higher (83.3 vs. 34.6%). Furthermore, in our study two of the five isolates harbouring *bla*_CTX-M-27_ gene belonged to the C1-M27 subclade. The first ESBL-producing invasive *E. coli* isolates belonged to the C1-M27 subclade were detected in 2015 in Hungary (unpublished data, the National Public Health Center). In 2018, according to an observational study of Jánvári et al., 44.6% (75/168) of invasive ESBL-producing *E. coli* isolates investigated at the National Public Health Center belonged to the ST131 clone, where the ratio of C2/H30Rx and C1-M27 was 1 to 0.8 [28].

In this study, ST131, including the C1-M27 (2 isolates) and the C2/H30Rx subclades (3 isolates) proved to be the dominant multidrug-resistant *E. coli* clone in invasive infections. The three ST131 C2/H30Rx isolates formed a relatively close cluster (≤ 6 allele differences), and this could suggest an undetected outbreak affected two hospital wards (Infectious Diseases ward, 1st Internal Medicine ward). However, based on the results of plasmid replicon analysis it was unlikely.

All isolates were resistant to ceftriaxone and cefotaxime. The isolates with CTX-M-15 ESBL were resistant to ceftazidime, while the CTX-M-27 expressing isolates remained susceptible, probably because these enzymes hydrolyze ceftazidime poorly. Moreover, the C2/H30Rx isolates showed resistance to tobramycin and gentamicin probably because they have AAC(3)-IIa aminoglycoside-modifying enzyme [29].

We found one isolate that belonged to the emerging ST1193 global high-risk clone [30]. To the best of our knowledge, this study revealed for the first time the presence of the ST1193 *E. coli* in Hungary. This clone has been widely spread in Asia [31, 32]. The ST1193 was the second most frequent ST (23.7% of 99 isolates) after ST131 (37.3%) among ciprofloxacin-resistant invasive *E. coli* isolates in the Tertiary Care University Hospital in Korea [33]. In Europe, it has been described in Germany where the rate of ESBL-producing *E. coli* ST1193 was 0.6% (3/495) among all *E. coli* infections [34]. In a South-West England study, 11 of 836 *E. coli* from urine samples belonged to ST1193 [35].

It has been known that the narrow-host-range IncF type plasmids have been evolved along with fluoroquinolone-resistant ST131 H30 clades. The F2:A1:B- plasmid is associated with the C2/H30Rx subclade, whereas the F1:A2:B20 plasmid is strongly associated with the C1/H30R subclade [5]. The presence of Col-like plasmids has been reported also in ST131, and in ST1193 *E. coli* clones, however, the exact role of these plasmids is not clear [6]. In our study, each of the isolates had various Inc-type (F, Q) replicons and except Ec5, they had a Col-type (pHAD28, BS512, 156, 8282) replicons as well. The C1-M-27 isolates carried the Group1 plasmid replicons (IncFII_1, IncFIA_2, IncFIB_20) and the reads of Ec4 isolate covered the Group1 reference plasmid to a higher breadth compared to Ec5 (96 vs. 72%). The C2/H30Rx isolates did not co-encode together the standard Group2 plasmid replicons (IncFII_2, IncFIA_1). The reads of Ec1 and Ec3 shared 70% coverage with F2:A1:B- Group2 plasmid and Ec2 shared 47%. Ec1 and Ec3 had identical replicons to the pU1 plasmid (pMLST: F1:A1:B16) [6]. The coverage of pU1 by Ec1 and Ec3 reads was 91 and 92%, respectively. These findings suggest that these two isolates harbored a similar plasmid like the pU1. The ST1193 isolate had different replicons (IncFII_4-like, IncFIB_10) than in Group1 and Group2 plasmids.

It is not completely understood, which key factors determine the successful dissemination of the ST131 C2/H30Rx and C1/H30R (C1-M27) clones worldwide, besides the antibiotic genes, but the virulence armament probably has an important part in it. The ST131 extraintestinal *E. coli* isolates have a wide range of virulence-coding genes, including adhesins, invasines, iron-uptake factors, protectins, toxins that enable them to bind to the host cells, invade the host tissues and avoid host defense systems [36, 37]. Several studies concerned that there are differences in the content of the ST131 virulence factors, and only a few virulence genes were consistently identified in all strains. The content can vary with their virotypes [24]. We identified 56–63 virulence-associated genes in our ExPEC isolates. Among the virulence set we found, some virulence genes were associated with ST131 isolates, like *sat, iha, iucD, ompT, usp* [38–40]. The ST1193 isolate also carried the *sat, iha, vat, iutA, usp* which were associated with ST1193 isolate [30, 32, 41]. The isolates carried siderophores which enable them to survive in a low-iron condition: aerobactin (*iucABCD*), yersiniabactin (*irp2*), and the Ec2 isolate also carried the salmochelin (*iroBCDEN*) operon. The Ec2 showed virotype B, while the other isolates of both subclades of ST131 showed virotype C. The global disseminated virotype C has been described as the most prevalent *E. coli* ST131 virotype [24, 42]. There is only a few data in the literature about significancy of the ST131 isolate with virotype B [24, 43]. Blanco et al. found that virotype B was significantly associated with a lower likelihood of symptomatic infection compared to virotype C [24].

It has been described that ST131 clones can transmit among human and animal hosts [44]. Based on a study from Israel, the CTX-M-27-producing ST131 subclones had a higher transmission rate than CTX-M-15- producing ST131 subclones [45]. At this number of isolates, we cannot estimate if the selection effect of the antibiotic consumption influences the dissemination of the ST131 subclades, especially the third-generation cephalosporins, because it needs further investigation.

## Conclusion

This study has several limitations, like small sample size and a two months time limit. However, it gives an insight into the incidence and genomic characteristics of ESBL-producing *E. coli* isolates in a Hungarian hospital. It highlights the dominance of the ST131 clones and the C1-M27 and C2/H30Rx subclone in invasive infections. Although, further national studies are needed with a larger collection of isolates belonging to the two subclades to determine the possible factors (virulence, antibiotic resistance) that can influence their dissemination.

## Data Availability

The data set supporting the results of this article are included within the article.

## Abbreviations

BSI: Bloodstream infection
cgMLST: Core genome multilocus sequence typing
CT: Cluster-type
ESBL: Extended-spectrum β-lactamase
EUCAST: European Committee on Antimicrobial Susceptibility Testing
ExPEC: Extraintestinal pathogenic *E. coli*
FQ-R: Fluoroquinolone resistance
MALDI-TOF: Matrix-assisted laser desorption ionization time-of-flight mass spectrometry
MIC: Minimum inhibitory concentration
MLST: Multilocus sequence typing
SRA: Sequence Read Archive
ST: Sequence typing
WGS: Whole-genome sequencing
VFDB: Virulence Factors Database
